# The Causes, Pathology, and Indications for Treatment, of Bowel Affections of Young Children

**Published:** 1875-10

**Authors:** N. S. Davis

**Affiliations:** Chicago


### EXAMINER.

---

Vol. XXXII. — OCTOBER, 1875. — No. 10.

---

### Original Communications.

### THE CAUSES, PATHOLOGY, AND INDICATIONS FOR TREATMENT, OF BOWEL AFFECTIONS OF YOUNG CHILDREN.

Read before the Chicago Society of Physicians and Surgeons.

By N. S. DAVIS, M.D., Chicago.

The very great mortality among children under three years of age, from affections of the alimentary canal, during the two warmest months of the year, has very properly attracted the attention of many, both in and out of our profession. And as there is an increasing desire on the part of the more enlightened citizens in our large cities, to mitigate the suffering and lessen the mortality among these little ones, it is very desirable that the efficient causes of the sickness and mortality should be well understood. For on this will depend the judiciousness and efficiency of the prophylactic and sanitary measures to be adopted for their benefit.

In looking over the statistics of mortality in this city, from June 1st, 1874, to Aug. 1st, 1875, we find the following results:

| | 1874. | | | | | | | 1875. | | | | | | |
|---|---|---|---|---|---|---|---|---|---|---|---|---|---|---|
| | June. | July. | Aug. | Sept. | Oct. | Nov. | Dec. | Jan. | Feb. | Mar. | Apr. | May. | June. | July. |
| Deaths over five years...... | 222 | 279 | 284 | 239 | 258 | 232 | 263 | 284 | 197 | 271 | 288 | 290 | 228 | 236 |
| Deaths under five years and over two. | 31 | 58 | 44 | 48 | 41 | 36 | 36 | 50 | 31 | 63 | 52 | 68 | 38 | 58 |
| Deaths under two years.... | 312 | 1123 | 841 | 517 | 261 | 162 | 146 | 209 | 215 | 216 | 323 | 218 | 263 | 874 |
| Total Mortality............ | 565 | 1460 | 1169 | 804 | 560 | 430 | 445 | 543 | 443 | 550 | 663 | 576 | 529 | 1168 |

It will be seen by the details of this table that the gross mortality in fourteen months, was 9905; and of this number 5680 were literally babies under two years of age; only 654 between two and five years; and 3571 over five years of age.

The mortality over five years of age is distributed pretty uniformly throughout the year, the lowest being 197 in February, and the highest 290 in May.

The same is true of the mortality between two and five years of age, the lowest being 31 in each of the months of February and June, and the highest 68 in May. The mortality of infants under two years, however, follows a very different rule; the lowest being 146 in December, and the highest 1123 in July, 1874, and 874 in July, 1875. Indeed, almost half of the entire mortality of infants is crowded into the months of July, August and September of each year. The increase is abrupt and rapid, as will be seen by comparing the numbers for June and July of each year. For instance, the deaths in June, 1874, were, under two years of age, 312, in July, 1123; in June, 1875, 263, and in July, 874. Or, to make the comparison still closer, the week ending June 26, 1875, gave 69 deaths under two years, while the next week, ending July 3rd, gave 140; the next, ending July 10th, 183; and that ending July 17th, 253, which was the highest for any week during the present summer. In 1874, the week ending June 20th, gave a mortality under two years, of only 48; the next week, ending June 27th, 104; that ending July 4th, 131; that ending July 11th, 250; and the next, ending July 18th, 281, which was the climax for that year. The decline of infant mortality after reaching its climax, about the middle of July, is more gradual than the access, and extends through August and September, reaching in October about the average for the remainder of the year.

If we turn again to the details of the mortality tables, we shall find nearly all of the increased infant mortality during July, August, and September, to be caused by affections of the alimentary canal. This is illustrated by the following table, made up from the returns to the Board of Health:

| | 1874. | | | | | | | 1875. | | | | | | |
|---|---|---|---|---|---|---|---|---|---|---|---|---|---|---|
| | June. | July. | Aug. | Sept. | Oct. | Nov. | Dec. | Jan. | Feb. | Mar. | Apr. | May. | June. | July. |
| Cholera Infantum | 71 | 604 | 393 | 209 | 34 | 5 | 1 | 1 | .... | .... | 14 | 7 | 41 | 403 |
| Cholera Morbus | 2 | 11 | 7 | 1 | 1 | .... | .... | .... | .... | .... | .... | .... | 1 | 12 |
| Diarrhœa | 6 | 74 | 81 | 45 | 22 | 7 | 4 | 6 | 3 | 2 | 13 | 3 | 18 | 86 |
| Dysentery | 6 | 47 | 47 | 29 | 20 | 3 | 1 | 1 | 4 | 2 | 8 | 1 | 9 | 45 |
| Total Bowel Affections | 85 | 736 | 528 | 284 | 77 | 15 | 6 | 8 | 7 | 4 | 35 | 11 | 69 | 546 |

It is thus seen that of 1796 deaths from diseases classed under the heads of cholera infantum, cholera morbus, diarrhœa, and dysentery, 1548 occurred during the months of July, August, and September, leaving only 248 for the other nine months of the year. Of the 1796 deaths at all ages, from bowel affections, 1339 are from cholera infantum alone. And of the 1339, there occurred in July 604, August 393, Sept. 209, making an aggregate of 1206 in these three months. Of the remainder, 71 occurred in June, and 34 in October, leaving only 28 in the other seven months of the year. The facts developed by this analysis of the mortality of our city for the past twelve or fourteen months, are in exact accordance with the facts of every year, and are equally illustrated by the mortality statistics of every city located in the middle and northern belts of the United States, east of the Rocky mountains.

Substantially, then, we may say that the bowel affections of children are strictly endemic and limited definitely to the warm season of the year. A disease, or group of diseases that recurs with so much regularity every year, and proves so destructive to infantile life, must have fixed and definite causes on which it depends. First among these causes we must place heat, or certain elevation of atmospheric temperature. Twenty-five years of observation in this city, have led me to regard the first week of continuous hot, summer weather, that occurs after the middle of June, in this locality, as developing the beginning of the bowel affections of children for that season, with as much certainty as any other event in nature. This is well illustrated by comparing the tables already given with the meteorological conditions as exemplified in the tables furnished for the Signal Service Bureau. For instance, in June, 1874, the weeks ending the 13th and 20th respectively, gave an average mean daily temperature of 63.6° and 72.4° F., and only 1 death from cholera infantum was reported during the first, and 3 during the second. But the next week, ending the 27th, gave a mean daily temperature of 80.3° F., and 40 deaths from this disease. Also in June, 1875, up to the 19th of the month, the highest daily average range of temperature for any one week was 64° F., and only 4 deaths from cholera infantum had occurred in the 19 days. But the next week ending June 26, the temperature averaged 70.4° F., and 13 deaths occurred from that disease. The effect of temperature is again shown by comparing the mortality and temperature of the same month for different years. Thus July, 1874, gave a mortality from cholera infantum of 604, and a mean daily temperature of 74.7° F., while July, 1875, gave only 403 deaths from that disease, and a mean daily temperature of 67.2° F. It is not merely the high average temperature that influences the mortality from these intestinal affections, but its continuance through the twenty-four hours, and the extremes of the seasons. For instance, during the week ending June 12th, 1875, the thermometer rose on the 11th, at 2 P. M., to 87° F., but at 9 P. M. it had fallen to 71°, and at 7 the next morning to 50°, and no death from cholera infantum during that week.

During the week ending June 26th, the temperature rose to 72° F., at 2 P. M. of the 22d, and continued above that point night and day until it reached 83° F., on the afternoon of the 24th, making three days and nights of continuous high temperature. And it was during these three days that cholera infantum commenced its work for this year, 13 deaths from it having been reported by the end of the week. Yet the maximum temperature of this week was 4° below the maximum of the week ending June 12th. But the tables of mortality do not give us the full data for judging of the effects of temperature in the production of disease; because many who are attacked in one week will not die until from one to six weeks afterwards. Hence, to judge correctly of the relation of certain atmospheric conditions to the prevalence of any given disease, we need a record of the date of attacks instead of the date of deaths. From the observations of many years, aided by some written records, I am satisfied that 90 per cent. of all the cases of cholera infantum and serous diarrhœa that occur during each year, have their beginnings during the first four weeks of high summer temperature; which, in this city, is usually between the 20th of June and the 20th of July. During the last two seasons I have taken great care to inquire concerning the date of the initial symptoms in all cases of bowel affections in children, coming under my observation, and I have very rarely found one in August or September that had not had its beginnings in July or the last week in June.

The same remark does not apply, however, to dysentery or ileo-colitis. I will go still further, and claim as the result of my inquiries, that four-fifths of all the attacks of cholera infantum and diarrhœa in young children develop their initial symptoms during those special periods when we have from two to five or six consecutive days and *nights* of high temperature, with stillness or only light winds and a high atmospheric moisture. Whether the morbid effects are induced by these conditions directly, or by the absence of ozone and electricity, which generally accompany the conditions named, must be left for further observation to determine.

While the atmospheric conditions just alluded to, constitute the efficient or determining causes of the disease under consideration, there are some collateral circumstances that greatly increase their efficiency. Of these, living in crowded, ill ventilated, uncleanly, and damp dwellings or localities, are of the greatest importance. To be satisfied of this, we need only to compare the ratio of infant mortality in different wards and sections of the same city. Or compare the ratio of mortality among the children of foreign born parents, with those of the native population. But all these collateral conditions exist as much at one season of the year as another, and hence it it is evident that the influence they exert is only secondary. The popular notion that a large part of the bowel affections, so destructive to infantile life, are induced by the growth of the first set of teeth irritating the gums, is sufficiently disproved by the statistical facts already given. I presume no one will pretend that there were not as many children "cutting teeth" in this city, during the months of February and March, of the present year, as in July; and yet not a death occurred from cholera infantum during the two former, while 403 were buried during the latter. That the imperfect development of the mucous membrane of the alimentary canal, and the extreme sensitiveness of the nervous structures during the first two years of life, render children during that period, extremely sensitive to the relaxing influence of high temperature combined with the coincident atmospheric conditions previously stated, is true ; and is sufficient to explain the great prevalence of the disease at that period of life. But that the simple growth of the teeth has any more influence than the growth of any other bone in the body, we have no reason to believe.

If cholera infantum, and the ordinary intestinal affections of young children, arise from the atmospheric conditions we have named, what is the modus operandi of these conditions? In other words, what is the essential pathology of these affections?

It is a well established fact that heat increases the *excitability* of living animal tissues, while by separating the organic atoms farther from each other, it diminishes the tonicity or force of vital affinity. When to this effect of temperature is added the depressing effect of a deficiency of ozone, that generally accompanies high temperature and high degrees of atmospheric moisture, we have just that combination of influences calculated to establish a morbid degree of sensitiveness coupled with loss of tone in the delicate mucous membrane of the alimentary canal in young children. These pathological conditions diminish the natural function of the membrane as an absorbing surface, and increase the tendency to exudation or effusion. Hence the frequent and thin discharges, varying in degree from only two or three per day, of a semi-fluid character, up to the most violent cholera morbus.

Though morbid sensitiveness and relaxation or loss of tonicity constitute the primary and essential pathological changes in these affections, yet others follow, such as general weakness, rapid loss of flesh, and in many cases the establishment of inflammatory action in portions of the mucous membrane. When this latter change takes place, there accompanies it more or less febrile action, and small mucous discharges, sometimes mixed with blood.

If what has been said in regard to the atmospheric conditions which determine the attacks of bowel affections in young children, is true, it is plain that all sanitary or prophylactic measures must have for their object an interruption of the high temperature of such seasons as we have described, or the counteraction of the effects on the living system. The most effectual methods of doing this, are the removal of the infants, either to an elevated, hilly or mountainous region, where the water would be pure, the air dry, and the nights cool; or to floating hospital ships, in which they may be carried out far enough from land to get the cooler and purer air of the sea or lake. To be effectual in *preventing* disease, these measures should be called into active operation coincidently with the first week of continuous warm weather, each year, and continued diligently until the first six weeks of high summer temperature have passed. If such measures are deferred until the middle of July, they will do very little in lessening the number of cases, because a very large majority of the cases will have already passed their initial stage. Yet they may still be of great service as curative agents in aiding to restore those already more or less sick. In cases not able to adopt either of the measures just named, the next most effectual preventive measures are free ventilation of the dwellings, and especially the sleeping rooms, judicious bathing, and whenever the temperature of the evening continues above 70° F., beyond 8 o'clock, P. M., let the abdomen and whole trunk of the body be enveloped in a towel wet in cool water, and left to dry out gradually during the night. Let the infants be taken out freely in the open air during the pleasantest part of each day. If the child begins to look pale, its flesh to feel soft and flabby, and the passages from the bowels to be slightly more frequent and fluid than natural, the following formula may be used with great benefit: ℞. Aromat. Sulph. Acid, ʒ ij; Tinct. Opii, ʒ j; Tinct. Cinchonæ, ℥ jss; Syr. Prun. Virgin., ℥ jss. M. From five to ten drops of this may be given in a teaspoonful of water, to a child under eight months of age. From ten to fifteen drops may be given when the child is between eight and eighteen months, and it may be repeated from one to three times a day. The indications for treatment after attacks have fairly commenced, are so directly deducible from the pathological conditions already stated, and I have, in former years, so often expressed my views of the best means for fulfilling those indications, that I will not repeat them here.

